# Supplementary material for: Climate impacts from North American boreal forest fires
Source: Nat Geosci. 2026 Mar 3;19(4):455–61. doi: 10.1038/s41561-026-01940-3 (PMC13079090; doi:10.1038/s41561-026-01940-3)
Supplement: Supplementary file 1 — Supplementary Figs. 1–14, Tables 1 and 2, Text, Methods and references. [file 41561_2026_1940_MOESM1_ESM.pdf]

nature geoscience

Supplementary information https://doi.org/10.1038/s41561-026-01940-3

# Climate impacts from North American boreal forest fires

In the format provided by the authors and unedited

**Table of contents**

Supplementary text

Supplemetry Methods

Supplementary Figures 1–14,

Supplementary Tables 1 and 2,

Supplementary references

## Supplementary information

## S1. Supplementary text

### S1.1 Temporal and spatial study domain

Radiative forcing is used to quantitatively compare the anthropogenic and natural drivers of climate change. The influence of a forcing mechanism on the climate is assessed by its impact on the net energy fluxes at the top-of-atmosphere, expressed in W m$^{-2}$ [1–4]. We applied this concept to assess the net impact of North American boreal forest fires between 2001 and 2019 on climate, in comparison to a no-fire situation. In the main text and supporting materials, radiative forcing estimates in units of W m$^{-2}$ of burned area, represent climate impacts over a 70-year post-fire period using shared socioeconomic pathway SSP2-4.5[5]. We constrained our spatial domain by the Arctic-Boreal Vulnerability Experiment's extended domain, known as the ABoVE domain. The ABoVE extended domain encloses the state of Alaska (USA), and provinces of the Yukon, Northwest Territories, British Columbia, Alberta, Saskatchewan, and Manitoba across Canada. Within this domain, we focused on fires that occurred between 2001 and 2019.

Our aim was to investigate the long-term climate impacts of boreal fires while ensuring the inclusion of accurate and comprehensive fire records. The timeframe was carefully selected to balance several considerations. Firstly, our study required fire records that surpass the temporal coverage of remote sensing databases, like those from Landsat, Sentinel-2 or MODIS. Hence, records from the Alaska Large Fire Database[6] and Canadian National Fire Database[7,8] form the foundation of this study. While these fire databases contain recorded fires since the 1920s, the earlier records were marked by greater inaccuracies and omissions. Additionally, most Canadian provinces did not begin comprehensive reporting of fires until the 1960s and 1970s. As a result, we limited our assessment to a 70-year post-fire period. Secondly, the 70-year period adequately captures the albedo changes following a fire and the subsequent return to pre-fire conditions[9,10] as vegetation regrows. This dynamic is particularly important, as albedo is a critical factor in the radiative forcing balance post-fire. However, it is important to note that the older fire scars in these fire databases may have included patches of vegetation that remained unburned within the fire perimeters.

### S1.2 Average climate impacts from Alaskan and Canadian fires

We found large spatial variability in the net radiative forcing across boreal North America. Continental gradients and spatial variability in net radiative forcing are influenced by two large-scale patterns. Firstly, greenhouse gas emissions from combustion are, per unit of burned area, higher in the northwestern and western part of the domain because of deeper burning into organic soils[11]. Our estimates suggest radiative forcing from combustion (i.e. greenhouse gases emitted at the time of fire) contribute to climate warming with an average of 9.51 ± 3.43 W m$^{-2}$ in Alaska and 8.78 ± 3.17 W m$^{-2}$ in Canada (Figure S10). Secondly, the surface albedo cooling effects from prolonged spring snow cover are stronger near the latitudinal treeline (boundary between the forest and tundra ecosystems), where snow exposure persists longer in spring[12] (Figure S13). Across boreal North America, the climate-cooling, driven by changes in post-fire surface albedo of -6.62 ± 1.06 W m$^{-2}$ (Alaska) and -8.71 ± 1.40 W m$^{-2}$ (Canada), offsets the climate-warming caused by direct greenhouse gas and precursor emissions to varying degrees, either partially or, in some cases, completely[13]. The post-fire greenhouse gas emissions from permafrost thaw follow a similar pattern to the forcing from combustion, albeit with a smaller radiative forcing magnitude. We estimated an average positive forcing of 1.40 ± 0.74 W m$^{-2}$ from fire-induced permafrost thaw in Alaska and 0.37 ± 0.19W m$^{-2}$ in Canada (Figure S11). Our radiative forcing estimates from aerosol emissions indicate a climate-cooling effect of approximately -3.38 ± 2.88 W m$^{-2}$ in Alaska and -2.73 ± 2.32 W m$^{-2}$ in Canada (Figure S13). Post-fire vegetation carbon sequestration has a small average negative forcing of -0.56 ± 0.04 W m$^{-2}$ in Alaska and -0.59 ± 0.05 W m$^{-2}$ in Canada

(Figure S12). The modest radiative cooling impacts from vegetation recovery reflects the climate system's greater sensitivity to the immediate, large pulse of $CO_2$ emitted by fire. This large impulse persists in the atmosphere for centuries, compared to the gradual carbon uptake by vegetation that occurs over decades. This temporal asymmetry means that the warming effect of emissions dominates the climate response, limiting the offsetting potential of post-fire carbon sequestration. Over a longer timeframe, the contributions would likely become more comparable, however other well-mixed greenhouse gas emissions may continue to play a significant role. It is important to emphasize that the forcing agents have different spatial and temporal footprints (Extended Figure 5). To demonstrate the temporal radiative forcing dynamics after a fire, we show the warming and cooling forcing from the 2009 Minto Flats South (Figure S9). The Minto Flats South fire burned roughly 216,000 ha between June 21st and August 12th of 2009. Here, the combined climate-warming effects of direct greenhouse gas and precursor emissions, along with permafrost greenhouse gas emissions, are shown in red and largely determine the net radiative forcing after the fire. In the first two decades, we observe a strong initial climate-cooling driven by aerosol emissions in the first year, which is then gradually overtaken by the effects of surface albedo changes and vegetation recovery (Figure S9).

**S1.3 Comparison of climate impacts of North American boreal forest fires with climate impacts from tundra fires**

We did not include tundra fires in our analysis. Our space-for-time methodology, which is used in the post-fire albedo and net ecosystem $CO_2$ exchange predictions, is heavily dependent on long-term fire perimeter databases from Alaska[6] and Canada[7,8]. This satellite-based approach works well in data-rich boreal forest regions due to their high suitability for satellite-based change detection. The techniques, however, need further optimization for tundra ecosystems[10]. Tundra fires do not provoke similar structural ecosystem changes as do forest fires. Strong structural changes after forest fires lead to strong biogeophysical snow albedo increases in spring, and thus strong regional climate-cooling. Hence, the climate-cooling impact from surface albedo changes after tundra fires is expected to be minimal [14,15]. In addition, tundra ecosystems are underlain by permafrost and fire-induced active layer thickening may result in additional greenhouse gas emissions in regions with carbon-rich soils[16]. Taken together, tundra fires are likely climate-warming fires, yet there may be considerable regional variability in responses depending on fire severity and permafrost conditions[17,18].

## S2. Supplementary methods

### S2.1 Radiative forcing from well-mixed greenhouse gases and precursors

We used carbon emissions from the Arctic-Boreal Vulnerability Experiment fire emissions database (ABoVE-FED) together with published emission factors to estimate the climate feedbacks from greenhouse gas and aerosol emissions[10,19–22]. In doing so, we used biome-specific emission factors to convert fire consumption of dry matter into emissions of individual greenhouse gas species. Based on these emission factors, we calculated the biome-specific dry matter carbon content (DMCC) required for the conversion of fuel consumption into emissions of individual species, which is 47.33%. This technique has been applied in fire inventories to estimate emissions stemming from various species. For Arctic-boreal fires, we derived emission factors from meta-analyses that show the mean values from different field campaigns[19,23]. Table S1 lists the emission factors and global warming potentials for different greenhouse gases and precursors used in our modeling approach. We assume that $CO_2$, CO, and $CH_4$ emissions represent the majority of fire carbon emissions.

**Table S1 | List of emission factors and global warming potentials for greenhouse gases, precursors, and aerosols.**

| | Emission factor[a] | Global warming potential (GWP) | |
|---|---|---|---|
| | | 20-year | 100-year |
| Carbon dioxide ($CO_2$) | 1530±140 | 1 | 1 |
| Methane ($CH_4$) | 5.5±2.5 | 80.8±25.2[b] | 27.2±11[b] |
| Nitrous oxide ($N_2O$) | 0.24±0.06 | Not used | Not used |
| Non-methane volatile organic compounds (NMVOC) | 6.6±4.9 | 16.2±9.2[c] | 5.0±3.0[c] |
| Carbon monoxide (CO) | 121±49 | 5.6±1.8[d] | 1.8±0.6[d] |
| Nitrogen oxide ($NO_X$) | 1.18±0.86 | -2.4±30.3[e] | - 8.2±10.3[e] |
| Black carbon (BC) | 0.43±0.21 | 2900±1500[f] | 830±440[f] |
| Organic carbon (OC) | 5.9±2.5 | -160±68[f] | -46±20[f] |

[a] All emission factors are given in kilogram species per kilogram dry matter combusted, following Andreae[19], Table 1 "Boreal forest", [b] following Forster et al.[24], Table 7.15 "GWP, $CH_4$-non-fossil", [c] following Fry et al.[22] and Collins et al.[20], Table 8.A.5 "GWP, VOC North America", [d] following Fry et al.[22], Collins et al.[20] and Myhre et al.[25], Table 8.A.4 "GWP, CO North America", [e] following Fry et al.[22], Collins et al.[20] and Myhre et al.[25], Table 8.A.3 "GWP, $NO_X$ North America", [f] following Bond et al.[26] and Myhre et al.[25] Table 8.A.6 "GWP, BC (aerosol-radiation interaction + albedo, global) and OC (global)".

We used the simplified expressions for radiative forcing of $CO_2$, $CH_4$ and $N_2O$ from Etminan et al.[1] (Equations 1-3). Fire-related $CO_2$ emissions were converted into concentrations estimated at yearly intervals after the impulse. The lifetime of $CO_2$ varies across different future emissions scenarios, contingent upon the effectiveness of ocean and land sinks[27,28]. We used the climate impulse-response functions for the computation of greenhouse gas metrics derived under different climate scenarios from Joos et al.[28]. These functions represent the fraction of a $CO_2$ impulse remaining in the atmosphere under future climate conditions for each post-fire year. The functions presented by Joos et al.[28] correspond to various background conditions as simulated with the BERN3D-Lund-Potsdam-Jena Dynamic Global Vegetation Model (BERN3D-LPJ) accounting for different Representative Concentration Pathways (RCP) climate scenarios. In this context, the behavior of the function is dependent on the magnitude of carbon emissions. We leveraged upon the concentration-dependency to estimate the response function for various Shared Socioeconomic Pathways (SSPs). Therein, the SSP response functions leverage upon the marginal increase in atmospheric concentrations between paired RCP and SSP climate scenarios. Therefore, the trajectories of the SSP response functions mirror

the trajectories derived from the BERN3D-LPJ model for the RCP scenarios. However, the RCP and SSP trajectories diverge from each other because of different atmospheric greenhouse gas concentrations up until 2100. The yearly $CH_4$ and $N_2O$ atmospheric concentrations were modeled with a lifetime-dependent pulse outflow model[29]. The lifetime of $CH_4$ was assumed to be fixed at 12.4 years, whereas the lifetime of $N_2O$ varies over time depending on future $N_2O$ concentrations. We incorporated this variability in lifetime with future ambient concentrations[17,30].

$$RF_{CO_2} = [a_1(C - C_0)^2 + b_1|C - C_0| + c_1\bar{N} + 5.36] \times \ln\left(C/C_0\right), \quad (1)$$

$$RF_{N_2O} = [a_2\bar{C} + b_2\bar{N} + c_2\bar{M} + 0.117](\sqrt{N} - \sqrt{N_0}), \quad (2)$$

$$RF_{CH_4} = [a_3\bar{M} + b_3\bar{N} + 0.043](\sqrt{M} - \sqrt{M_0}), \quad (3)$$

$$\bar{N} = 0.5 \times (N + N_0), \quad (4)$$

$$\bar{M} = 0.5 \times (M + M_0), \quad (5)$$

Constants:
$a_1$= -2.4×10$^{-7}$ (W m$^{-2}$ ppm$^{-1}$), $b_1$= 7.2×10$^{-4}$ (W m$^{-2}$ ppb$^{-1}$), $c_1$= -2.1×10$^{-4}$ (W m$^{-2}$ ppb$^{-1}$),
$a_2$= -8.0×10$^{-6}$ (W m$^{-2}$ ppm$^{-1}$), $b_2$= 4.2×10$^{-6}$ (W m$^{-2}$ ppb$^{-1}$), $c_2$= -4.9×10$^{-6}$ (W m$^{-2}$ ppb$^{-1}$),
$a_3$= -1.3×10$^{-6}$ (W m$^{-2}$ ppb$^{-1}$), $b_3$= 8.2×10$^{-6}$ (W m$^{-2}$ ppb$^{-1}$)

Where, in these functions C, N and M represent the future atmospheric $CO_2$, $N_2O$ and $CH_4$ concentrations because of fire-disturbance. While $C_0$, $N_0$ and $M_0$ relate to the ambient concentrations in an undisturbed state for a given year under SSP2–4.5[5].

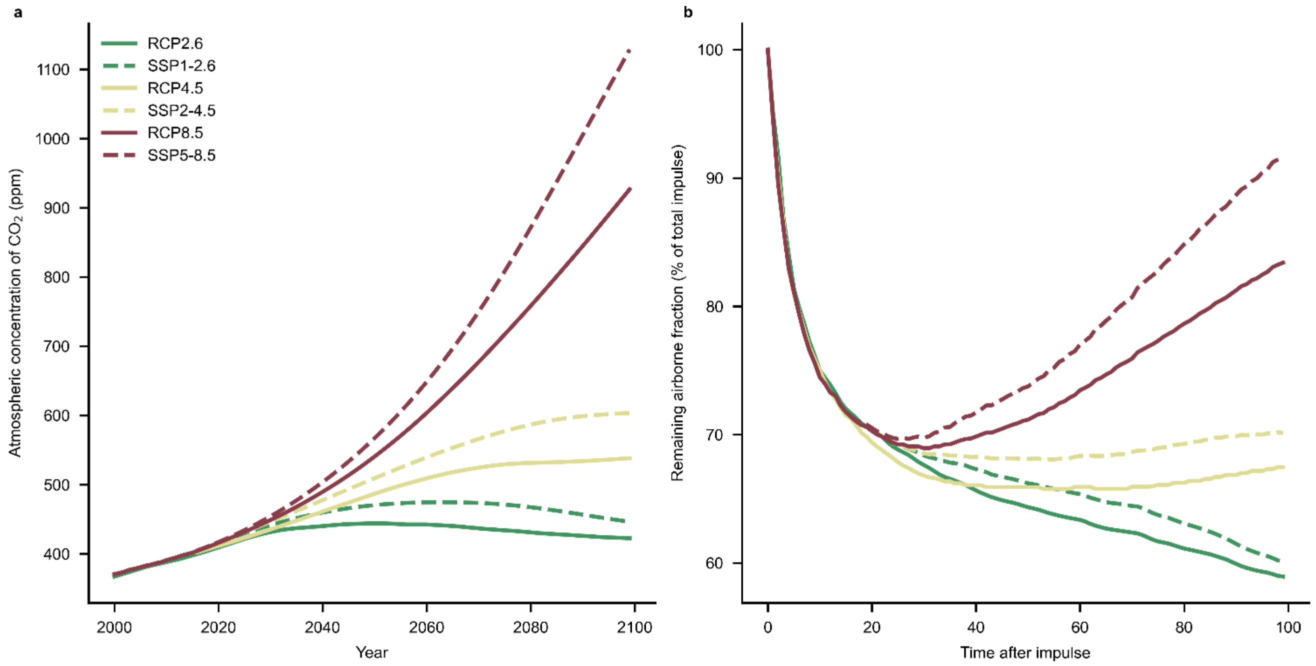


**Figure S1 | Atmospheric concentrations and airborne fraction of CO2.** (a) Atmospheric concentration under future climate scenarios in parts per million derived from Meinhausen et al., (2020)[5]. (b) Impulse response function for different background conditions under different future scenarios (solid lines, reference function for Representative Concentration Pathways (RCP) climate scenarios as simulated with the Bern3D-Lund-Potsdam-Jen model by Joos et al.[28], dashed-lines represent functions of Shared Socioeconomic Pathways (SSP) climate scenarios).

We modeled the radiative forcing of precursors by interpolating between global warming potentials (GWPs) (Table S1) following Moubarak et al.[17]. In our approach, we linked the radiative forcing of precursors to the trajectory of $CH_4$, considering their prolonged impact on $CH_4$[20]. For CO and NMVOCs,

we assumed that the climate-warming effect within the initial 20-years remains constant, subsequently aligning with the trajectory of $CH_4$. To achieve this, we multiplied the relative warming ratio ($R_{[t][q]}$) for each trace gas (as defined in Equation 6) by the decay trajectory of cumulative radiative forcing for $CH_4$ to estimate its relative warming effect. In the relative warming ratio equation, the Global Warming Potentials (GWP) and emissions factor (EF) are defined per gas $_{[q]}$ for a given GWP time horizon $_{[t]}$. Finally, we derived the cumulative radiative forcing from CO and NMVOC as a function of the RF from $CH_4$ using the estimated relative warming ratio of CO and NMVOC to $CH_4$. On short timescales, $NO_X$ shows a positive global warming potential, while on long-term timescales, it shows a negative global warming potential. Therefore, we used an exponential decay ratio, which was parameterized by a non-linear least squares function to compute the relative warming effect of $NO_X$ to $CH_4$. To accomplish this, we adopted the approach outlined in Moubarak et al.[17], which involves calculating the relative warming ratio for $NO_X$ at year one after the fire. This is achieved by scaling the relative warming ratio in accordance with the cumulative radiative forcing response from $CH_4$ over the first 20-years after the fire. We computed the cumulative radiative forcing from $NO_X$ as a function of the RF from $CH_4$ using its estimated relative warming ratio:

$$R_{[t][q]} = \frac{GWP_{[t][q]} * EF_{[q]}}{GWP_{[t][CH4]} * EF_{[CH4]}}, \quad (6)$$

### S2.2 Radiative forcing from aerosol emissions

The impact of aerosol emissions on radiative forcing occurs shortly after a fire, as aerosols are typically removed from the atmosphere through wet and dry deposition within a few weeks. Nevertheless, we computed the cumulative mean over the entire 70-year period following the methods described in Moubarak et al.[17]. Our study considers both the direct and indirect forcing of black carbon (BC) and organic carbon (OC) aerosols. Firstly, we estimated the relative warming ratio of BC and OC to $CH_4$ following Equation 6. However, considering the short-lived nature of aerosols, we assessed the relative warming ratio based on the 20-year global warming potential (Table S1). To account for the indirect radiative forcing from aerosol, we used the ratio of indirect cloud effect in comparison to the direct effect under all-sky conditions. The direct-to-indirect conversion ratio used in this study was derived from Ward et al.[29].

### S2.3 Radiative forcing from changes in surface albedo after a fire

We used random forest regression models[31] to model post-fire surface albedo based on environmental and climate variables. For each model, we used MODIS-derived monthly mean blue-sky albedo as the target variable. We established monthly mean blue-sky albedo for January through December filtered by various quality flags from a daily mean short wave blue-sky albedo across North America at 500 m resolution between 2000 and 2017[32] derived from the MODIS MCD43A1 V006 Bidirectional Reflectance Distribution Function and Albedo (BRDF/Albedo)[33]. For spring and summer months (May through September), we included pixels produced with full inversions of best or good quality and aerosol optical depth (AOD) measurement derived from the MOD08 V6 Atmosphere Daily and Monthly Global Product Bands or the Multi-angle Imaging Spectro Radiometer (MISR) Level 3 Component Monthly Global Aerosol Product (MIL3MAE). For January, February, March, April, October, and November, we constrained our selection to the best or good quality MCD43A1 pixels, while disregarding all AOD requirements. Low sun angles in the month of December restricted us from deriving any observations. We thus averaged the monthly mean values of January and November as an estimate for December. A sensitivity analysis for these constraints is described in Potter et al.[10].

We trained separate random forest regressor models for each month and then for each post-fire year within that month using a suite of historical environmental and climatological features. The full list of input variables is provided in Table S2. To optimize computational efficiency and improve the

interpretability of feature importance, we applied feature selection to simplify each model by eliminating features outside of the top ten most influential predictors, based on their overall mean permutation feature importance across all models (Table S2). This approach allowed us to focus on the most relevant predictors while maintaining robust model performance. We trained random forest regressor models using the scikit-learn package in Python[34], with 50 trees and a maximum depth of 3. Each model was trained separately for post-fire years using an 80/20 train-test split, and performance was evaluated using $R^2$ and RMSE.

The predictive performance of the model was notably strong, as evidenced by the high correlations between the observed and predicted post-fire surface albedo values. The $R^2$ values ranged from 0.78 to 0.94 (Figure S2), indicating that a substantial portion of the variance in the observed data was explained by the model. This suggests that our post-fire albedo models were able to capture key patterns and trends in surface albedo following fire events. Additionally, the root mean squared errors fell between 0.01 and 0.06 (Figure S2), reflecting low levels of prediction error and confirming the model's accuracy in estimating post-fire albedo. In spring, represented here by the month of April, the most influential variables were related to evaporation and temperature, likely due to their impact on snow melt, soil moisture, and canopy dynamics. In summer, represented by the months June, July and August, soil characteristics become more important, likely due to their role in controlling heat retention and drainage, which affects the growth, density, and composition of vegetation. In early winter, temperature becomes particularly important because it directly influences the rate of snow accumulation and the timing of freeze-thaw cycles, which in turn affect surface albedo. Lower temperatures promote the formation of a stable snowpack, which has a high albedo and reflects more sunlight, while fluctuating temperatures can lead to melting and refreezing, altering the snow's reflectivity and surface properties (Figure S3).

**Table S2 | List of environmental and climatological variables used for the post-fire surface albedo and net ecosystem exchange (NEE) predictions.** Variables that were retained after feature elimination for each framework are indicated by the model names Albedo and NEE in superscript. If a climatological variable does not list a season, it represents the annual mean.

| Variable | Unit | Source |
|---|---|---|
| Percentage of clay | % | Hengl et al.[35] |
| Percentage of sand | % | Hengl et al.[35] |
| Percentage of silt | % | Hengl et al.[35] [Albedo] |
| Percentage of coarse | % | Hengl et al.[35] |
| Bulk density | g $cm^{-3}$ | Hengl et al.[35] |
| Soil organic carbon stock | tons $ha^{-1}$ | Hengl et al.[35] |
| Soil organic carbon content | g $kg^{-1}$ | Hengl et al.[35] |
| Soil water capacity until wilting point | Volumetric Fraction | Hengl et al.[35] |
| Soil water pH | pH | Hengl et al.[35] [Albedo] |
| Elevation | Meters | Danielson and Gesch[36] [Albedo] |
| Aspect | Degrees | Danielson and Gesch[36] |
| Slope | Degrees | Danielson and Gesch[36] |
| Topographic wetness index | Unitless | Danielson and Gesch[36] |
| Permafrost zonation index | 0-1 | Gruber[37] [Albedo] |
| Surface ruggedness index | 0-1 | Gruber[37] [Albedo / NEE] |
| Mean annual temperature | °C | Mahony et al.[38] |
| Mean temperature warmest month (MTWM) | °C | Mahony et al.[38] |
| Mean temperature coldest month (MTCM) | °C | Mahony et al.[38] |
| Temperature continentality (MTCM-MTWM) | °C | Mahony et al.[38] [NEE] |
| Mean annual precipitation | mm | Mahony et al.[38] |
| Mean summer precipitation (May to September) | mm | Mahony et al.[38] |
| Annual heat moisture index (MAT+10)/(MAP/1000) | Unitless | Mahony et al.[38] |
| Summer heat moisture index (MWMT/(MSP/1000) | Unitless | Mahony et al.[38] |
| Degree-days below 0 °C | Days | Mahony et al.[38] |
| Degree-days above 5 °C | Days | Mahony et al.[38] |
| Degree-days below 18 °C | Days | Mahony et al.[38] |
| Degree-days above 18 °C | Days | Mahony et al.[38] [NEE] |
| Number of frost-free days | Days | Mahony et al.[38] |
| Frost-free period | Days | Mahony et al.[38] |
| Start of frost-free period | Julian date | Mahony et al.[38] |
| End of frost-free period | Julian date | Mahony et al.[38] |
| Precipitation as snow | mm | Mahony et al.[38] |
| Extreme maximum temperature over 30-years | °C | Mahony et al.[38] |
| Extreme minimum temperature over 30-years | °C | Mahony et al.[38] [NEE] |
| Hargreave's reference evaporation | mm | Mahony et al.[38] [Albedo / NEE] |
| Hargreave's climate moisture index | mm | Mahony et al.[38] [Albedo] |
| Mean annual solar radiation | MJ $m^2$ $d^{-1}$ | Mahony et al.[38] |
| Hogg's climate moisture index | mm | Mahony et al.[38] |
| Degree-days above 10 °C and below 40 °C | Days | Mahony et al.[38] |
| Winter mean temperature (December to February) | °C | Mahony et al.[38] [NEE] |
| Spring mean temperature (March to May) | °C | Mahony et al.[38] [Albedo / NEE] |
| Summer mean temperature (June to August) | °C | Mahony et al.[38] |
| Autumn mean temperature (September to November) | °C | Mahony et al.[38] [Albedo / NEE] |
| Winter mean precipitation (December to February) | mm | Mahony et al.[38] |
| Spring mean precipitation (March to May) | mm | Mahony et al.[38] [NEE] |
| Summer mean precipitation (June to August) | mm | Mahony et al.[38] |
| Autumn mean precipitation (September to November) | mm | Mahony et al.[38] [Albedo / NEE] |

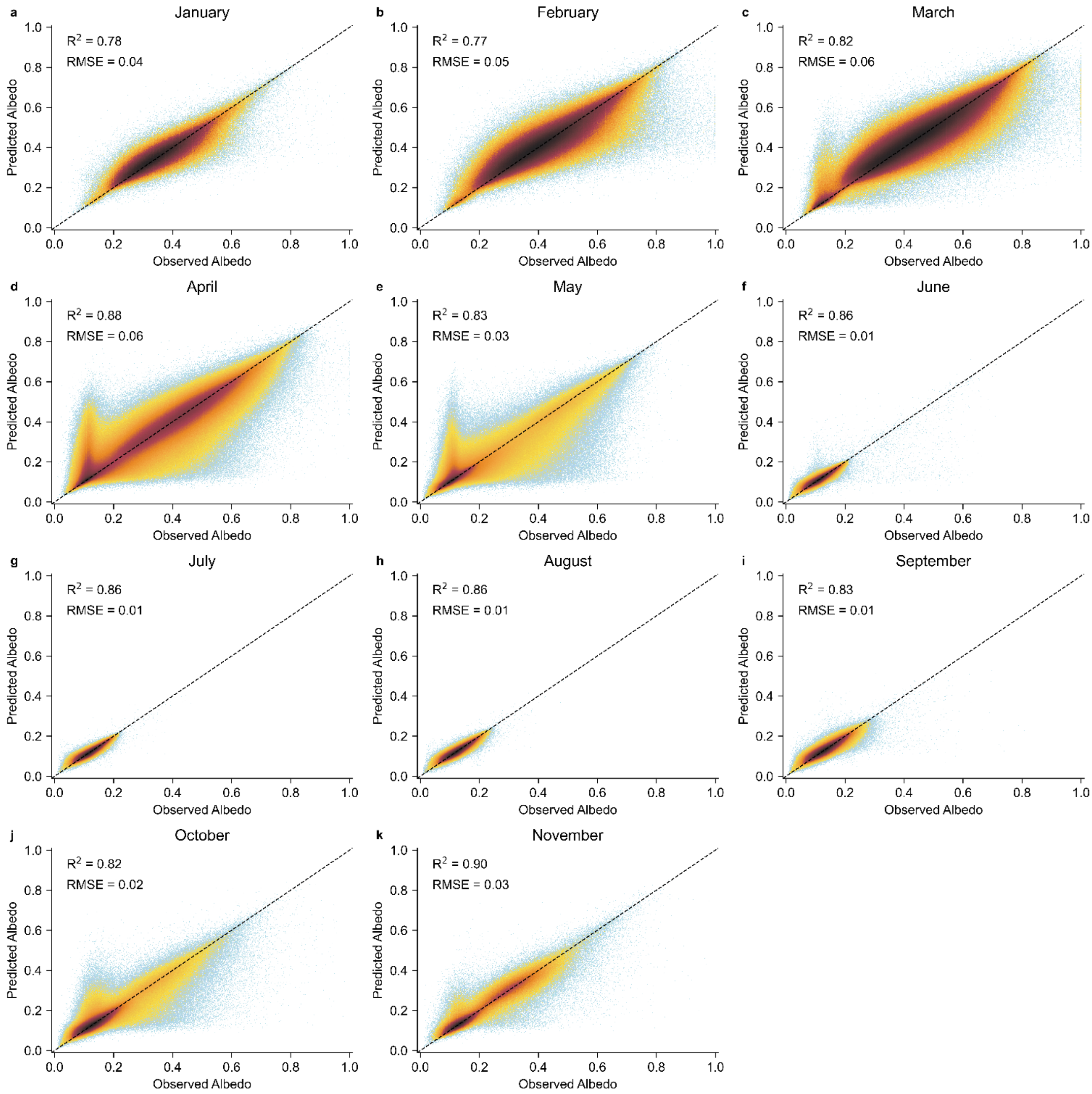


**Figure S2 | Monthly 2D histogram plots of observed vs. predicted post-fire albedo values and associated $R^2$ and root mean squared error (RMSE).** Post-fire albedo values for December were not modeled and are hence not shown here. Performance was evaluated based on linear regression fits using the test set during cross-validation. The color shading indicates pixel density across five hundred histogram bins. The dashed line is the 1:1 line.

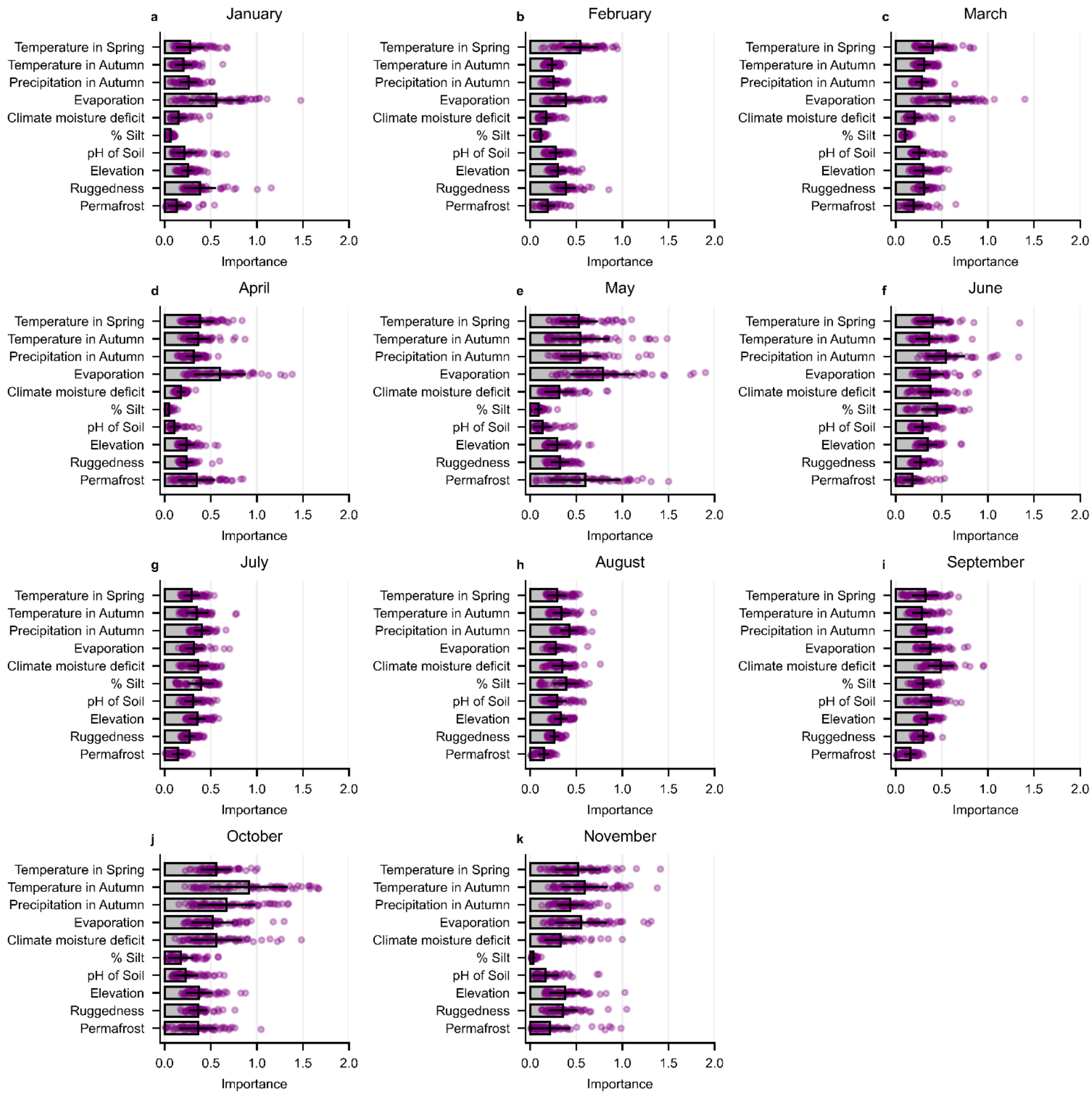


**Figure S3 | Variable importance plots for the ten most important predictors of the post-fire surface albedo random forest regressor models.** The variable importance bars show the mean scores across all 70-year periods for a given month. Error bars show one standard deviation of the mean variable importance scores. Individual model scores (n = 70) are shown in purple. December is not shown here as this month was modeled as the mean between the predicted albedo values in January and November.

**S2.4 Radiative forcing from vegetation recovery after a fire**

We chose the monthly NEE product from Virkkala et al.[39], which was upscaled from flux tower and chamber data, because it provides spatially explicit NEE data over our study domain, whereas towers and chambers may represent very localized conditions[40]. In addition, this NEE product performs reasonably well in capturing post-fire carbon dynamics[41]. Their analysis demonstrated that, while their product effectively represented the long-term time series of sites with recent fire history, its predictive performance varied when tested through cross-validation. The models trained with and without disturbed sites showed no significant differences in predictive accuracy[41]. Both the in situ flux observations and modeled NEE estimates follow the same increasing pattern after fire. However, it is important to note that the models do exhibit biases in burned areas towards underestimating carbon sources. This is likely because permafrost emissions are currently included through several indirect mechanisms rather than a direct representation. The NEE model from Virkkala et al.[39,41], included only 7% of the training data from burned forests on permafrost terrain, of which more than 75% are in isolated (0-10% coverage) and discontinuous (10-50% coverage) permafrost terrain. Hence fire-related NEE impacts tied to permafrost thaw are underrepresented and fluxes from active layer thickening responding to fire are likely missed. The underestimation bias is critical because permafrost soils store vast amounts of carbon, that can release substantial amounts of $CO_2$ and $CH_4$. Although the existing model effectively captures vegetation and upper-soil processes through broader environmental predictors[41], the dataset has likely limited sensitivity to permafrost thaw emissions. The dataset estimates post-fire NEE dynamics reasonably well after fire[41], nonetheless underestimates net $CO_2$ emissions in fire disturbed sites, particularly during the initial post-fire years (up to 7–10 years). This limitation contributes to documented underestimations, where carbon sources after fire are underestimated by up to 75 g C $m^{-2}$ $month^{-1}$, with an average of 5.2 ± 23.1 g C $m^{-2}$ $month^{-1}$ [41]. This is why we introduced a more direct integration of fire-induced permafrost emissions to provide a more comprehensive understanding of post-fire carbon budget (See Methods and supplementary section 2.5).

Our permafrost modeling framework estimates that 83 percent of all fire-induced permafrost emissions occur within the first two decades after the fire, totaling on average roughly 2.6 ± 1.2 kg C $m^{-2}$ as $CO_2$ over this period. This translates to counterbalancing a persistent net $CO_2$ bias of approximately 10.9 ± 5.1 g C $m^{-2}$ $month^{-1}$ in the existing NEE models over a 20-year timeframe. This estimate is approximately twice the magnitude of underestimations reported by Virkkala et al.[41], yet these values likely remain conservative. We could not incorporate post-fire subsidence and abrupt thaw events due to data and knowledge limitations. Turetsky et al.[42] estimated that abrupt thaw contributes a radiative forcing comparable to that from gradual thaw, effectively doubling the total permafrost-related climate impact when both processes would be considered.

We trained separate random forest regressor models for each post-fire year using a suite of historical environmental and climatological features (Table S2). To optimize our models and improve the interpretability of feature importance, we applied feature selection by eliminating features outside of the top ten most influential predictors (Table S2; Figure S5). This approach allowed us to focus on the most relevant predictors while maintaining robust model performance. We trained random forest regressor models using the scikit-learn package in Python[34], with similar tuning as done for the post-fire surface albedo predictions with 50 trees and a maximum depth of 3. We cross-validated each model separately using an 80/20 train-test split, and performance was evaluated using $R^2$ and RMSE (Figure S4). The stripe plots in Figure S4c and S4d show high correlations between the observed and predicted post-fire NEE values. The $R^2$ values ranged between 0.86 and 0.95 and root mean squared errors varied between 9.52 and 13.26 g C $m^{-2}$ $year^{-1}$ (Figure S4).

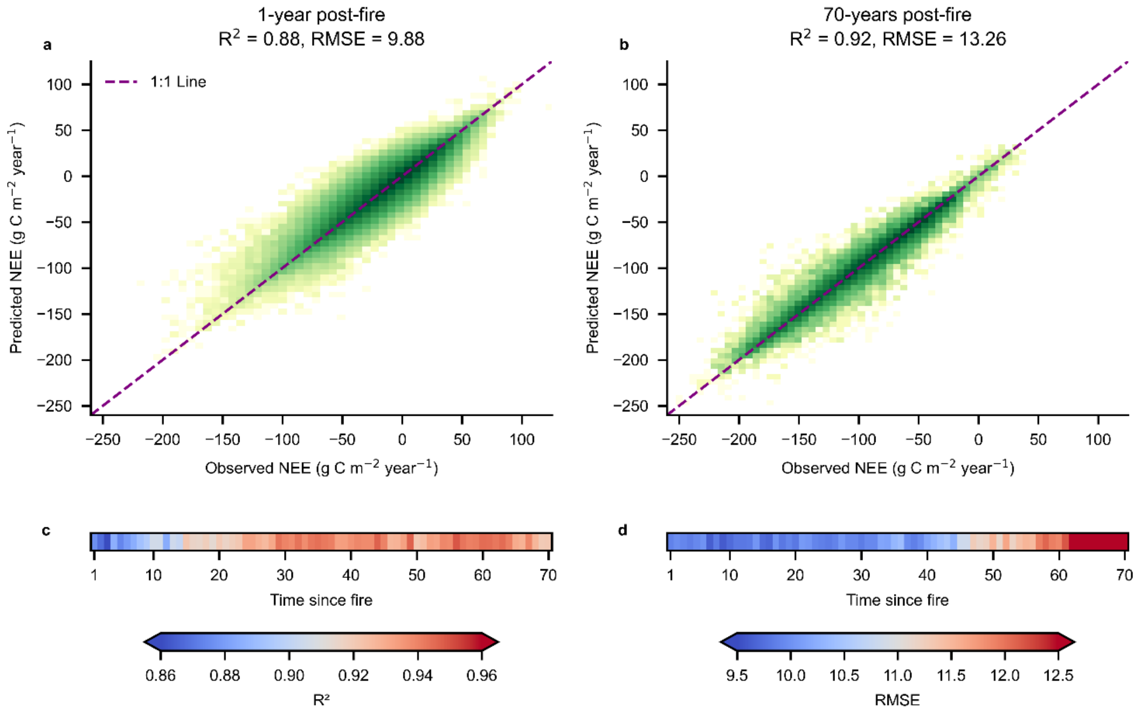


**Figure S4 | 2D histogram plot of observed vs. predicted post-fire net ecosystem exchange (NEE, g C m$^{-2}$ year$^{-1}$) values and associated $R^2$ and root mean squared error (RMSE) for 1-year post-fire (a) and 70-year post-fire (b).** Performance was evaluated based on linear regression fits using the test set during cross-validation. Color shading indicates pixel density across 50 histogram bins. The dashed line is the 1:1 line. Stripe plot of the associated $R^2$ (c) and RMSE (d) per post-fire year.

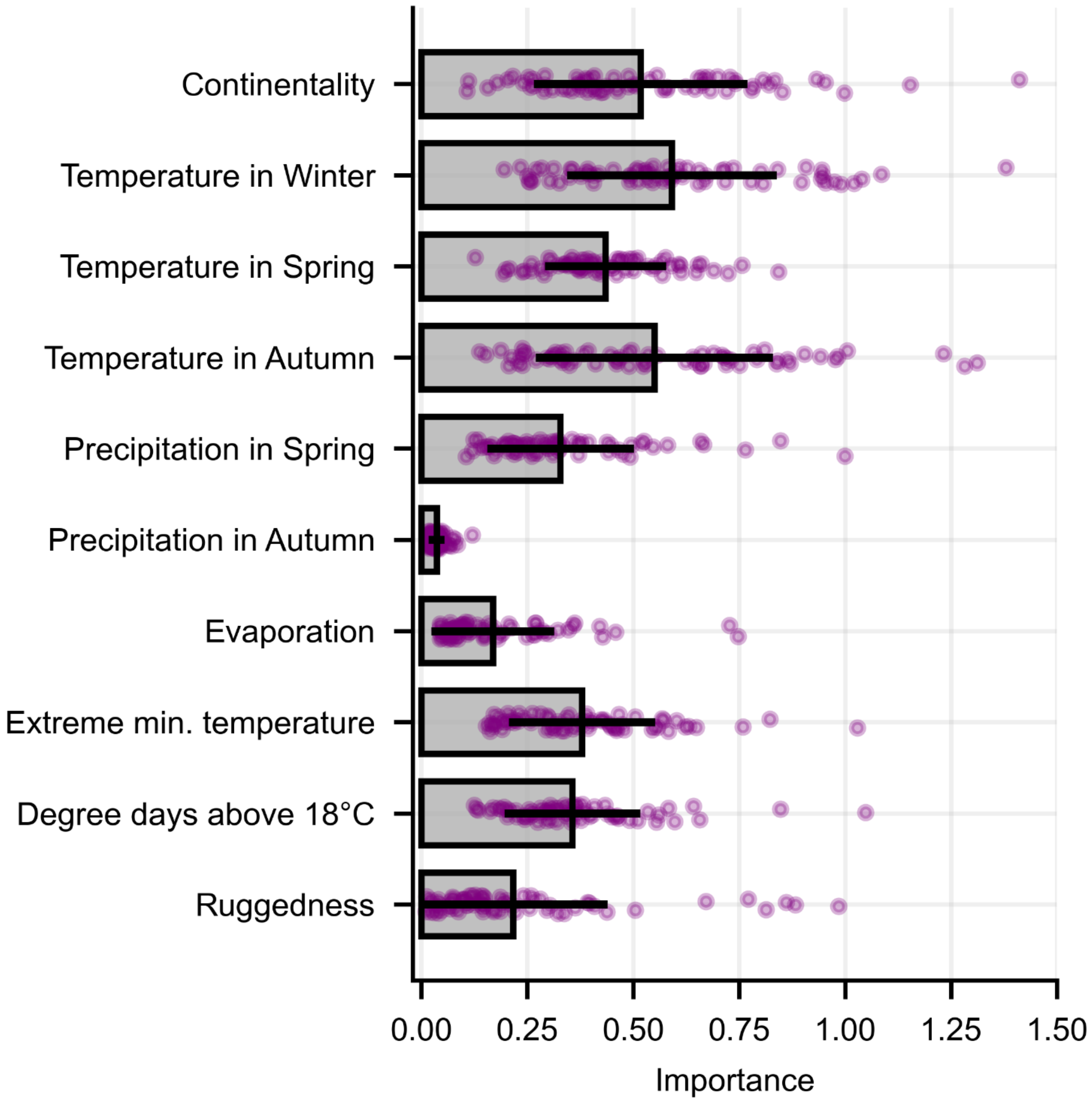


300

301 **Figure S5 | Variable importance plots for the ten most important predictors of the post-fire net ecosystem**
302 **exchange (NEE, g C $m^{-2}$ $year^{-1}$) random forest regressor models.** The variable importance bars show the mean
303 scores across all 70-year periods. Error bars show one standard deviation of the mean variable importance scores.
304 Individual model scores (n = 70) are shown in purple.

305

### S2.5 Fire-induced permafrost emissions modeling

In our estimates of the permafrost-carbon feedback after a fire, we used field observations from numerous studies to constrain the post-fire changes in active layer thickness (dALT). Here, we present the dALT as a percentage of the pre-fire ALT[43,44]. To create a continuous representation over a 70-year period, we fit an exponential regression model over time (Figure S6a), with an $R^2$ of 0.79 and RMSE of 8.57%. Additionally, we used carbon release curves after thaw for organic and mineral soils[45]. Schuur et al.[45] presented cumulative carbon release as a percentage of total carbon up to 10-years of aerobic incubations at a constant temperature of 5 °C. Their results show that soils from various locations within the permafrost region demonstrated comparable levels of potential microbial degradation of organic carbon after thaw, with emissions ranging between 1% and 76%. We used the averages for organic (*n = 43*) and mineral soils (*n = 78*) and fit a saturated growth model to predict the cumulative carbon release up to 70 years. By using the integral of the predicted cumulative release curves, we derived annual estimates of carbon releases for mineral and organic soils (Figure S6b). However, laboratory incubations provide a controlled environment and isolate the effect of temperature on microbial respiration. The controlled conditions in the lab may therefore lead to an overestimation of temperature sensitivity. To account for the overestimation in lab environments relative to field conditions, we included a scaling factor of 2.93[46] to correct the carbon release curves. This was calculated by comparing the temperature sensitivity $CO_2$ emissions based on in situ measurements (2.9, 95% CI = [2.1, 4.2]) and laboratory incubations (8.5, 95% CI = [5.0, 14.5])[46]. This scaling approach provides a more realistic estimate of carbon emissions from post-fire permafrost thaw under natural conditions.

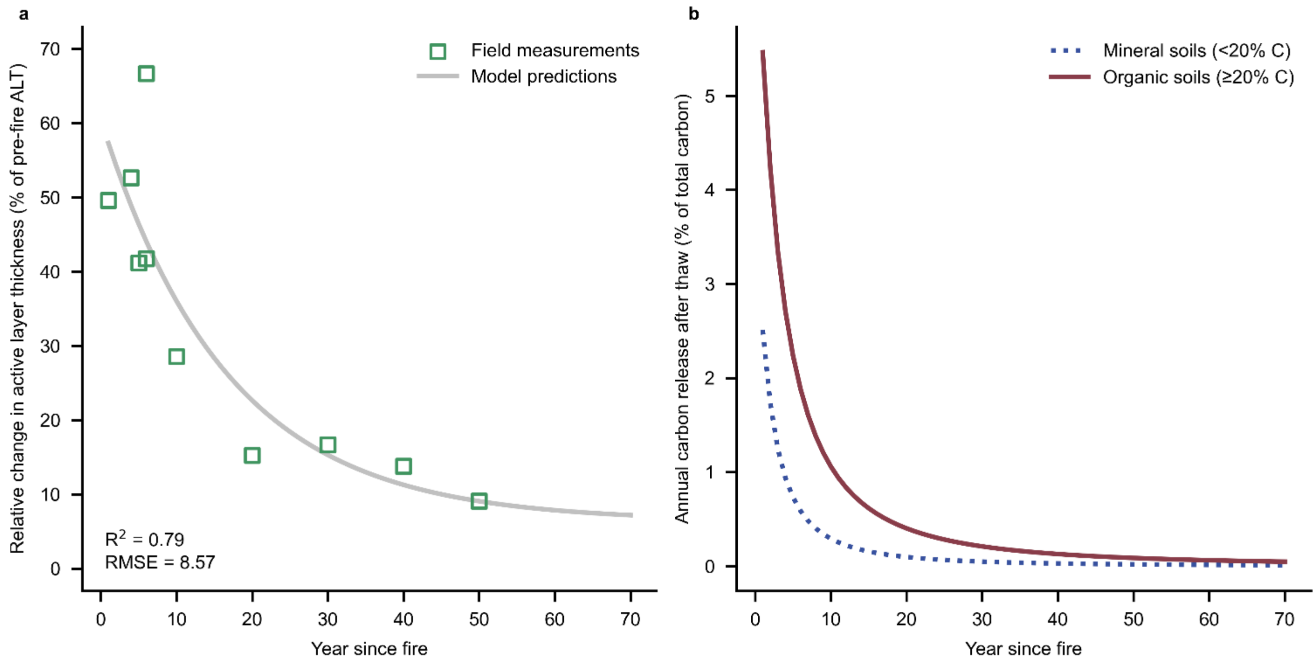


**Figure S6 | (a) Relative change in active layer thickness as percentage of the pre-fire active layer thickness.** Green squares represent various in situ field measurements derived from literature[43,44], gray line shows model predictions up to 70-year post-fire using a saturated growth model. (b) Annual carbon release curves after thaw as a percentage of total carbon for mineral soils (blue dotted line) and organic soils (red solid line)[45].

Based on the relative change in active layer thickness as a percentage of the pre-fire active layer thickness (Figure S4), we estimated the amount of soil carbon vulnerable to fire-induced thaw. This vulnerable carbon estimate was multiplied with the time dependent carbon release curve and a seasonality factor for the number of frost-free days within a given year to estimate the permafrost-carbon emissions after a fire. In addition, in our spatial framework, these estimates were multiplied by a fractional value between 0 and 1, which represented the likelihood of permafrost presence[37]. In

addition, we approximated the partitioning of carbon emissions into $CO_2$ and $CH_4$ emissions. In doing so, we used the $CH_4$:C ratio of 15.89% from Heffernan et al.[47] to calculate part of the carbon that will be emitted as $CH_4$, while we assumed that the remaining carbon will be emitted as $CO_2$. These emission estimates were transformed into radiative forcing estimates using the simplified radiative expression (Equations 1-5, supplementary section 2.1).

We validated our post-fire permafrost emissions using in situ rates of aged carbon release as $CO_2$ for a six[48] and nine[49] year post-fire environment. In these studies, radiocarbon ($^{14}C$) measurements from chamber-based post-fire soil respiration were used to distinguish between modern plant-derived and aged permafrost carbon. For the six-year post-fire environment, Estop-Aragonés et al. (2018a)[48] reported aged $CO_2$ emissions of 0.41 ± 0.20 g $CO_2$-C $m^{-2}$ $d^{-1}$. With a spatial and temporal matching, our model yields a comparable estimate of 0.50 ± 0.24 g $CO_2$-C $m^{-2}$ $d^{-1}$. Using data from Estop-Aragonés et al. (2018b)[49], representative for a nine-year post-fire environment, we found a strong agreement between the flux of 0.21 ± 0.06 g $CO_2$-C $m^{-2}$ $d^{-1}$ measured in the field and matching our modeled estimate of 0.23 ± 0.11 g $CO_2$-C $m^{-2}$ $d^{-1}$. These comparisons show that our model estimates of post-fire permafrost emissions are consistent with field measurements.

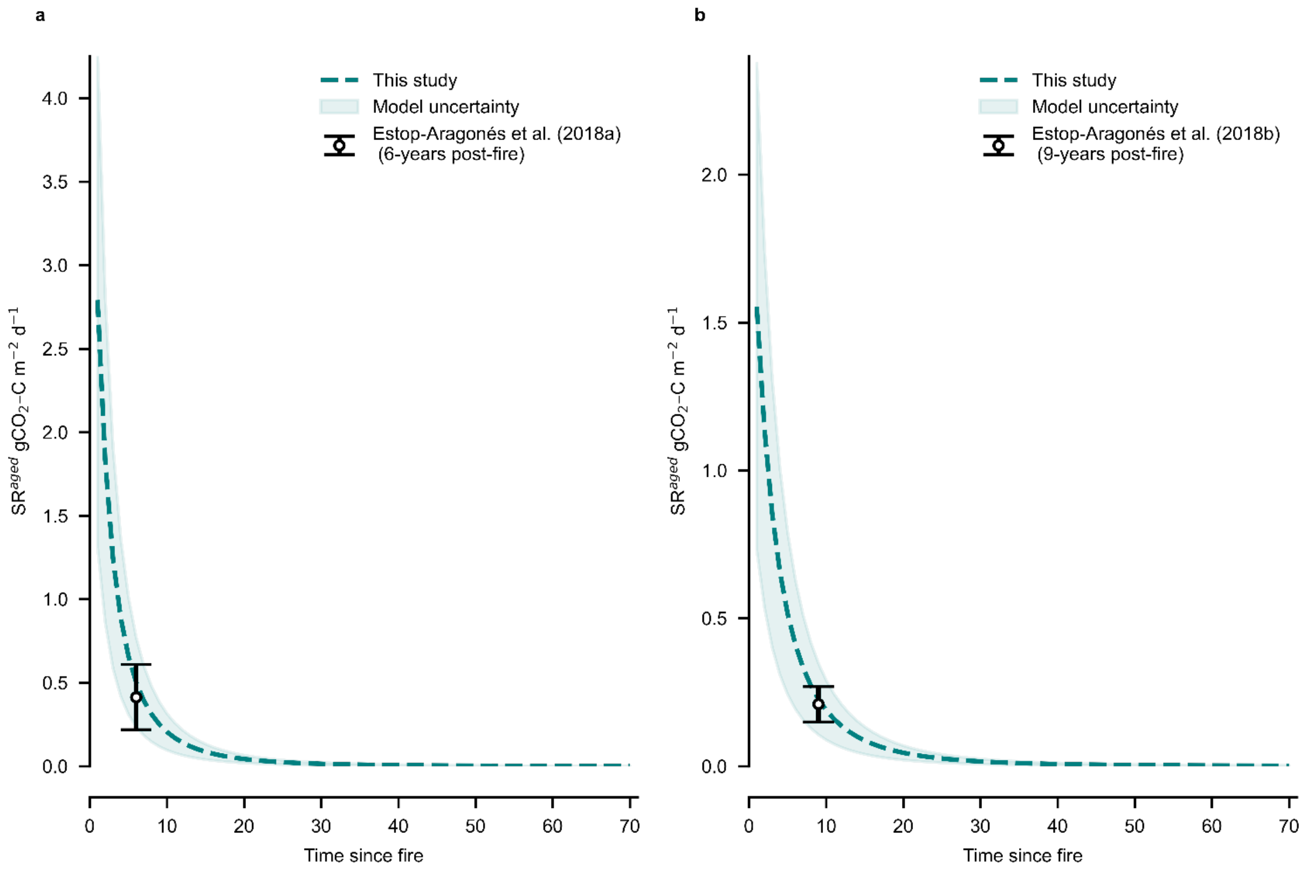


**Figure S7 | Validation of modeled soil respiration fluxes from post-fire permafrost thaw against in situ data derived from radiocarbon dated chamber flux measurements.** (a) Comparison of modeled and in situ permafrost carbon fluxes six-years post-fire in g $CO_2$-C $m^{-2}$ $d^{-1}$ based on Estop-Aragonés et al. (2018a)[48]. (b) Comparison of modeled and in situ permafrost carbon fluxes nine-years post-fire in g $CO_2$-C $m^{-2}$ $d^{-1}$ based on Estop-Aragonés et al. (2018b)[49]. Points represent the in situ $CO_2$-C emissions, with the bars representing the uncertainty as one standard deviation. The teal dotted lines show the model estimates, while the teal shading represents the model uncertainty based on the permafrost sensitivity framework. The model data presented here are spatially aligned with the coordinates from the two field measurements.

## S3. Supplementary figures

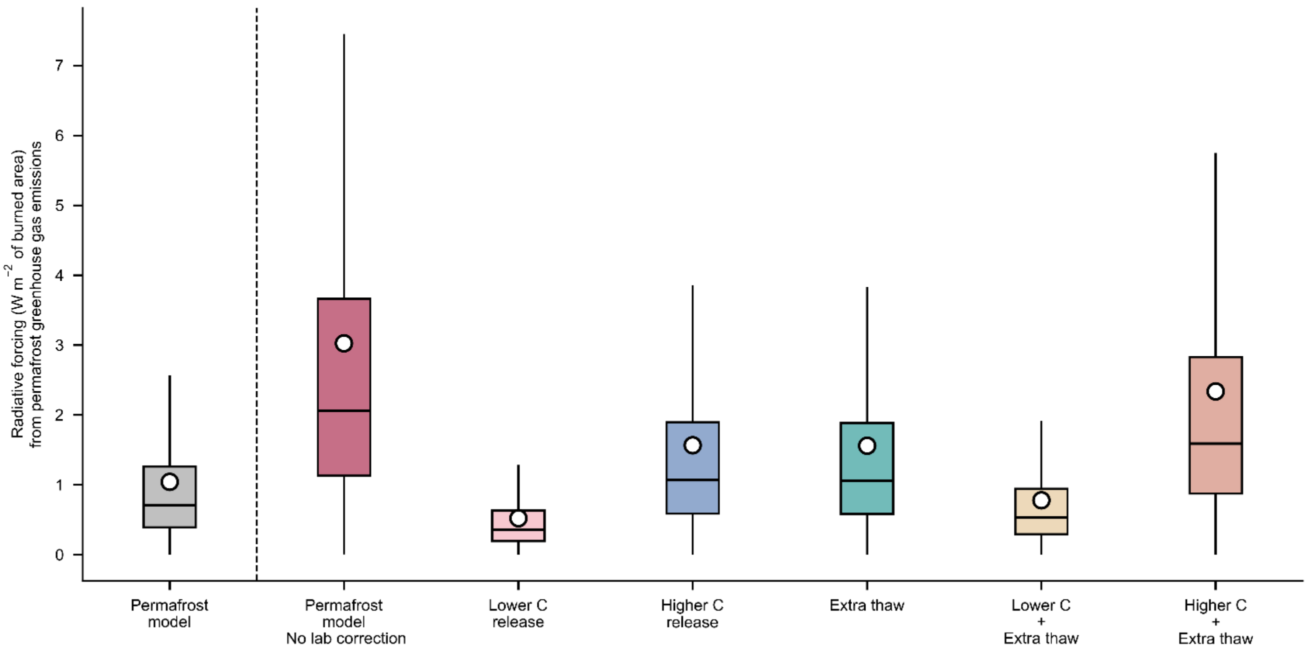


**Figure S8 | Radiative forcing from greenhouse gas emissions after fire-induced permafrost thaw.** The permafrost model, represented in gray, displays the range of estimates presented in our study, while the other boxplots represent different scenarios from a sensitivity analysis. The red boxplots show model estimates without applying a lab-to-field scaling factor of 2.93 based on Natali et al[46]. The pink boxplot corresponds to a 50% reduction in C release, while the blue boxplot represents an increase of 50% in emissions. The green boxplot represents an increase in thaw depth by 50%. The yellow and orange boxplots show the combined effects of changes in emissions and additional thaw. Each boxplot represents n = 10,000 burned pixels randomly selected from the dataset. Horizontal lines represent the median, the white circle represents the mean and upper and lower limits of the boxes show the 25$^{th}$ and 75$^{th}$ percentiles. Whiskers extend up to 1.5 times the interquartile range.

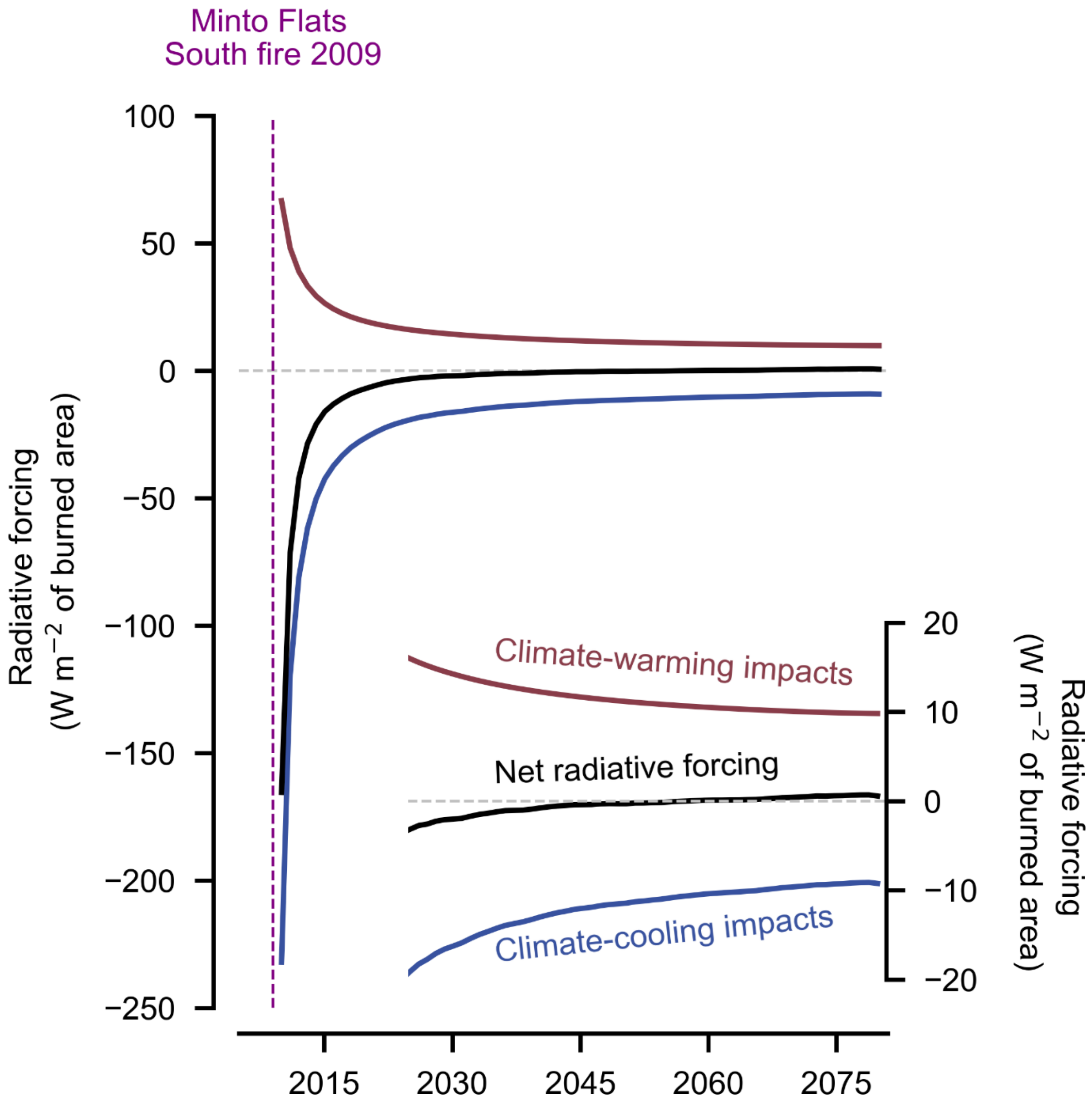


**Figure S9 | Positive, negative and net climate radiative forcings from the 2009 Minto Flats South fire over time.** The Minto Flats South fire burned approximately 216,000 ha between June 21st and August 12th of 2009. Climate-warming impacts are shown in red and represent the combined impact of direct greenhouse gas and precursor emissions and permafrost greenhouse gas emissions. Climate-cooling impacts, in blue, represent the combined impact from post-fire changes in surface albedo, aerosol emissions, and post-fire vegetation recovery. The inset shows the transition from an initial climate-cooling effect to a net climate-warming impact over time.

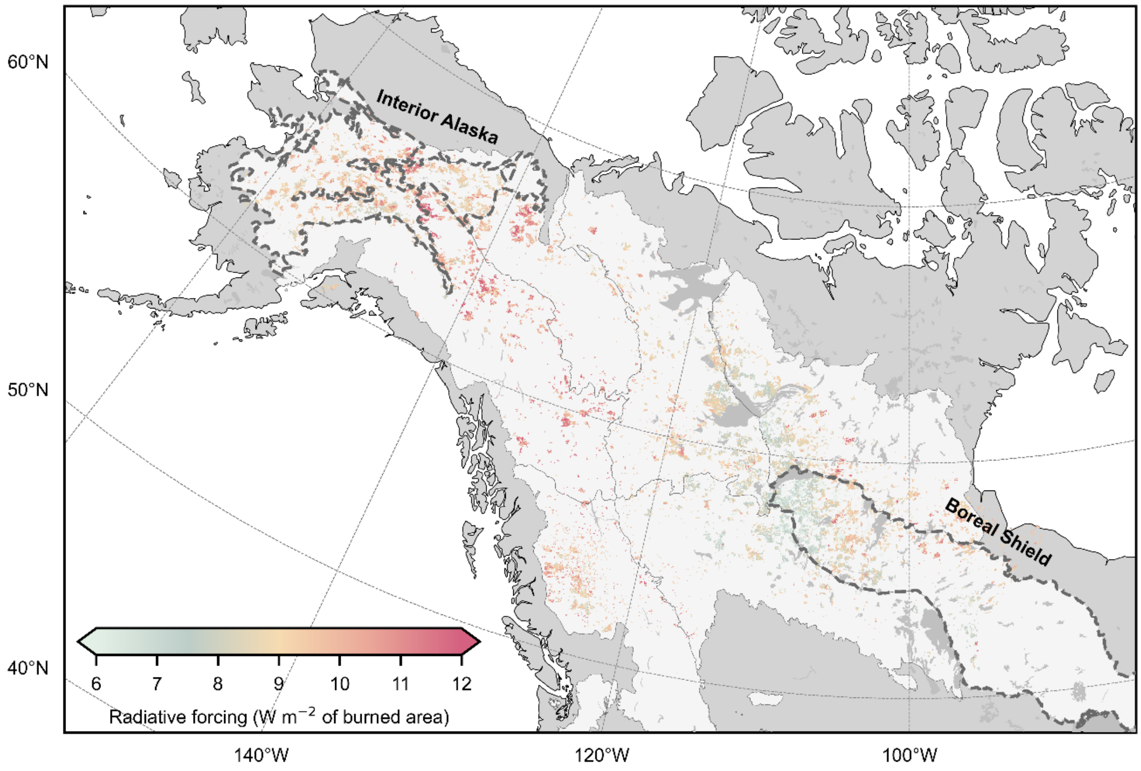


**Figure S10 | Cumulative mean climate radiative forcing from direct greenhouse gas and precursor emissions from fires between 2001 and 2019 across Alaska and western Canada over a 70-year period.** The ecoregion boundary for Interior Alaska and the Boreal Shield are delineated by the dashed gray lines. Ecoregion data were obtained from the United States Environmental Protection Agency (EPA) (https://www.epa.gov/eco-research/ecoregions-north-america, last accessed 21 January 2026). Basemap in figure is made with Natural Earth.

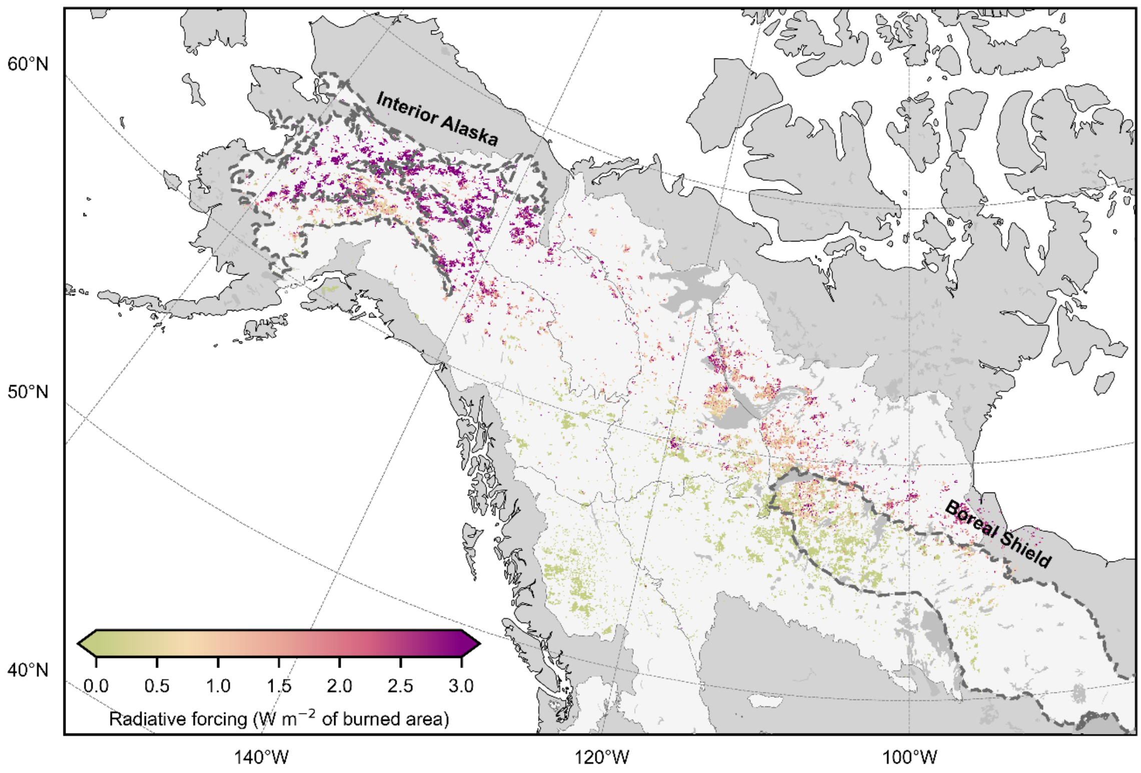


**Figure S11 | Cumulative mean climate radiative forcing from permafrost greenhouse gas emissions from fires between 2001 and 2019 across Alaska and western Canada over a 70-year period.** The ecoregion boundary for Interior Alaska and the Boreal Shield are delineated by the dashed gray lines. Ecoregion data were obtained from the United States Environmental Protection Agency (EPA) (https://www.epa.gov/eco-research/ecoregions-north-america, last accessed 21 January 2026). Basemap in figure is made with Natural Earth.

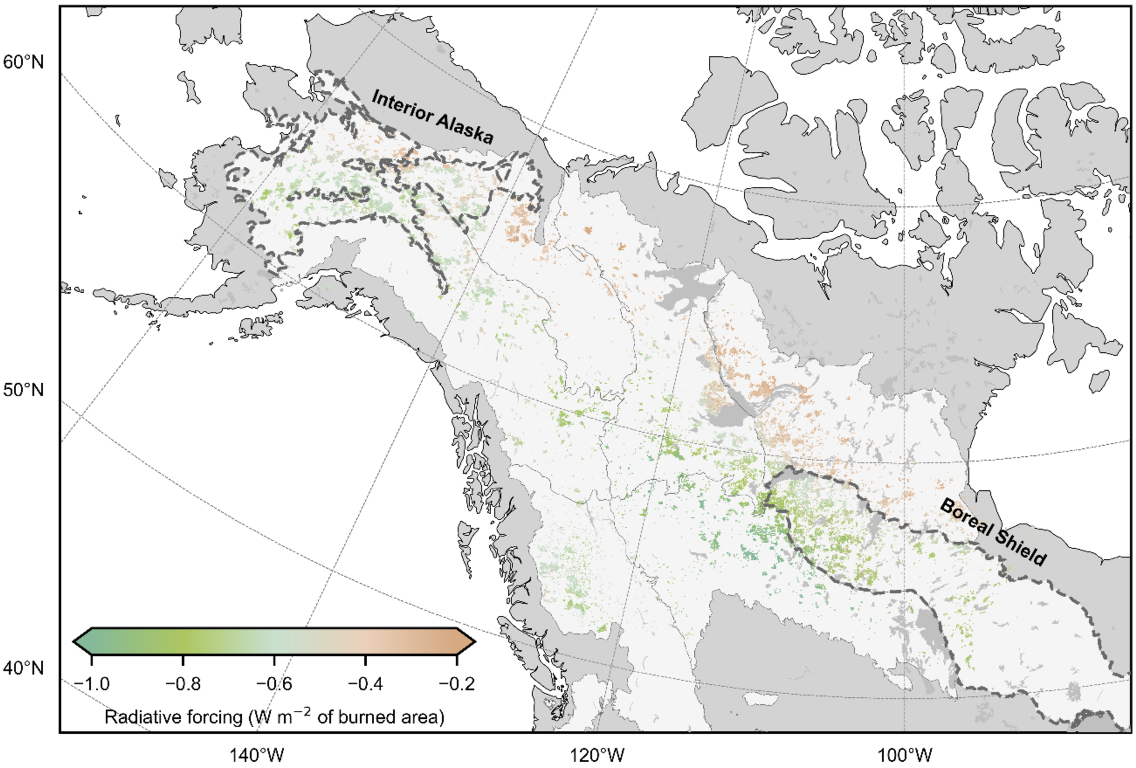


**Figure S12 | Cumulative mean climate radiative forcing from post-fire vegetation recovery from fires between 2001 and 2019 across Alaska and western Canada over a 70-year period.** The ecoregion boundary for Interior Alaska and the Boreal Shield are delineated by the dashed gray lines. Ecoregion data were obtained from the United States Environmental Protection Agency (EPA) (https://www.epa.gov/eco-research/ecoregions-north-america, last accessed 21 January 2026). Basemap in figure is made with Natural Earth.

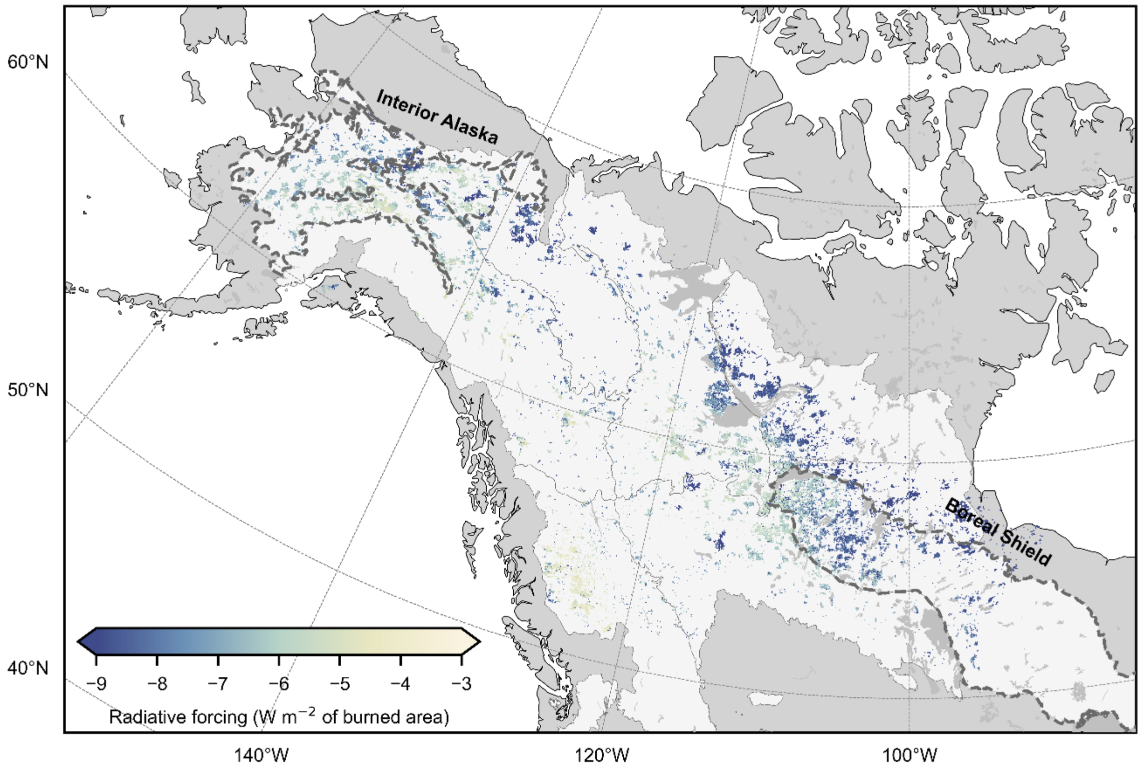


**Figure S13 | Cumulative mean climate radiative forcing from post-fire changes in surface albedo from fires between 2001 and 2019 across Alaska and western Canada over a 70-year period.** The ecoregion boundary for Interior Alaska and the Boreal Shield are delineated by the dashed gray lines. Ecoregion data were obtained from the United States Environmental Protection Agency (EPA) (https://www.epa.gov/eco-research/ecoregions-north-america, last accessed 21 January 2026). Basemap in figure is made with Natural Earth.

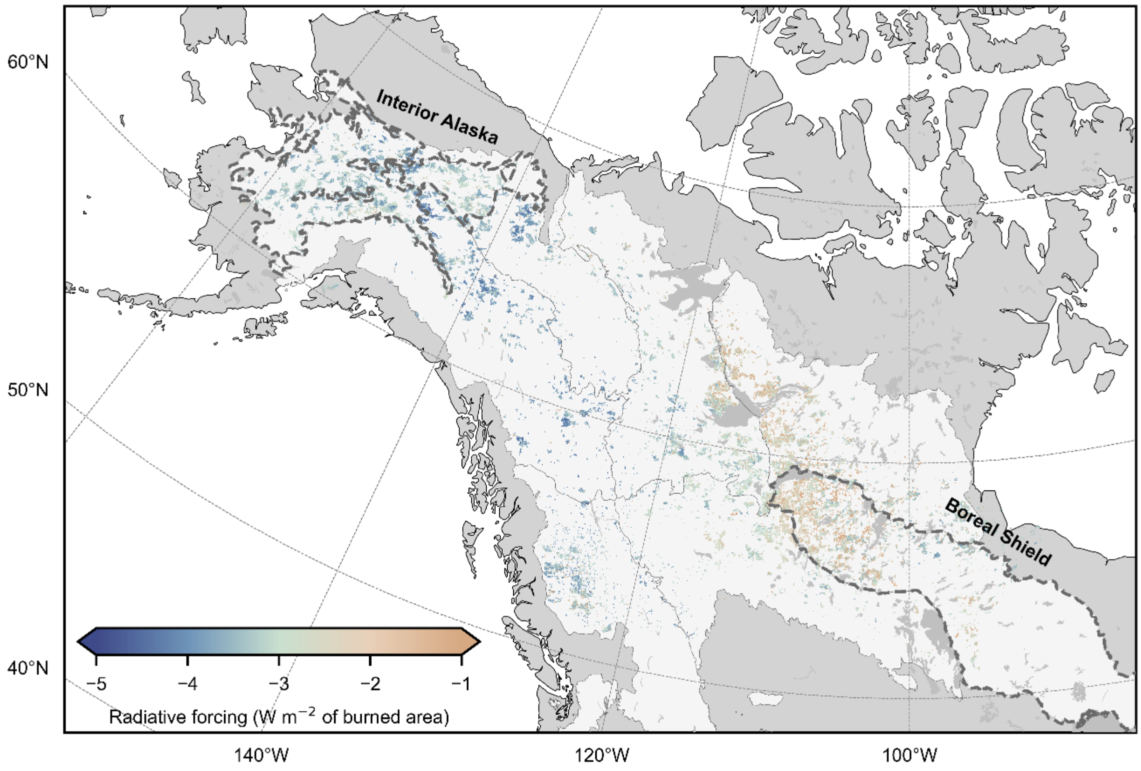


**Figure S14 | Cumulative mean climate radiative forcing from aerosol emissions from fires between 2001 and 2019 across Alaska and western Canada over a 70-year period.** The ecoregion boundary for Interior Alaska and the Boreal Shield are delineated by the dashed gray lines. Ecoregion data were obtained from the United States Environmental Protection Agency (EPA) (https://www.epa.gov/eco-research/ecoregions-north-america, last accessed 21 January 2026). Basemap in figure is made with Natural Earth.

## S4. Supplementary references


1. Etminan, M., Myhre, G., Highwood, E. J. & Shine, K. P. Radiative forcing of carbon dioxide, methane, and nitrous oxide: A significant revision of the methane radiative forcing. *Geophys. Res. Lett.* 43, 614–623 (2016).
2. Flanner, M. G., Shell, K. M., Barlage, M., Perovich, D. K. & Tschudi, M. A. Radiative forcing and albedo feedback from the Northern Hemisphere cryosphere between 1979 and 2008. *Nat. Geosci.* 4, 151–155 (2011).
3. Soden, B. J. *et al.* Quantifying climate feedbacks using radiative kernels. *J. Clim.* 21, 3504–3520 (2008).
4. Myhre, G., Highwood, E. J., Shine, K. P. & Stordal, F. New estimates of radiative forcing due to well mixed greenhouse gases. *Geophys. Res. Lett.* 25, 2715–2718 (1998).
5. Meinshausen, M. *et al.* The shared socio-economic pathway ( SSP ) greenhouse gas concentrations and their extensions to 2500. 3571–3605 (2020).
6. Kasischke, E. S., Williams, D. & Barry, D. Analysis of the patterns of large fires in the boreal forest region of Alaska. *Int. J. Wildland Fire* 11, 131–144 (2002).
7. Stocks, B. J. *et al.* Large forest fires in Canada, 1959-1997. *Journal of Geophysical Research: Atmospheres* 108, (2002).
8. Amiro, B. D. *et al.* Direct carbon emissions from Canadian forest fires, 1959-1999. *Canadian Journal of Forest Research* 31, 512–525 (2001).
9. Randerson, J. T. *et al.* The impact of boreal forest fire on climate warming. *Science (1979).* 314, 1130–1132 (2006).
10. Potter, S. *et al.* Climate change decreases the cooling effect from postfire albedo in boreal North America. *Glob. Chang. Biol.* 26, 1592–1607 (2019).
11. Potter, S. , *et al.* Daily Burned Area and Carbon Emissions Across Western Boreal North America between 2001 and 2019. *Biogeosciences* 20, 2785–2804 (2023).
12. Hessilt, T. D. *et al.* Geographically divergent trends in snow disappearance timing and fire ignitions across boreal North America. *Biogeosciences* 21, 109–129 (2024).
13. Hasler, N. *et al.* Accounting for albedo change to identify climate-positive tree cover restoration. *Nat. Commun.* 15, (2024).
14. French, N. H. F., Whitley, M. A. & Jenkins, L. K. Fire disturbance effects on land surface albedo in Alaskan tundra. *J. Geophys. Res. Biogeosci.* 121, 841–854 (2016).
15. Rocha, A. V. *et al.* The footprint of Alaskan tundra fires during the past half-century: Implications for surface properties and radiative forcing. *Environmental Research Letters* 7, (2012).
16. Heijmans, M. M. P. D. *et al.* Tundra vegetation change and impacts on permafrost. *Nature Reviews Earth and Environment* vol. 3 68–84 Preprint at https://doi.org/10.1038/s43017-021-00233-0 (2022).
17. Moubarak, M., Sistla, S., Potter, S., Natali, S. M. & Rogers, B. M. Carbon emissions and radiative forcings from tundra wildfires 1 in the Yukon-Kuskokwim River Delta, Alaska 2. *Biogeosciences* https://doi.org/10.5194/bg-2022-144 (2023) doi:10.5194/bg-2022-144.

18. Jones, B. M. *et al.* Post-fire stabilization of thaw-affected permafrost terrain in northern Alaska. *Sci. Rep.* 14, (2024).
19. Andreae, M. Emission of trace gases and aerosols from biomass burning. Global Biogeochemical. *Atomospheric Chemistry and Physics* 15 (4), 955–966 (2019).
20. Collins, W. J. *et al.* Global and regional temperature-change potentials for near-term climate forcers. *Atmos. Chem. Phys.* 13, 2471–2485 (2013).
21. Forster, P. M. *et al.* Chapter 7: The Earth's Energy Budget, Climate Feedbacks, and Climate Sensitivity. *Climate Change 2021: The Physical Science Basis. Contribution of Working Group I to the Sixth Assessment Report of the Intergovernmental Panel on Climate Change* in press (2021).
22. Fry, M. M. *et al.* The influence of ozone precursor emissions from four world regions on tropospheric composition and radiative climate forcing. *Journal of Geophysical Research Atmospheres* 117, (2012).
23. Akagi, S. K. *et al.* Emission factors for open and domestic biomass burning for use in atmospheric models. *Atmos. Chem. Phys.* 11, 4039–4072 (2011).
24. Forster, P. *et al.* The Earth's Energy Budget, Climate Feedbacks and Climate Sensitivity. in *Climate Change 2021: The Physical Science Basis. Contribution of Working Group I to the Sixth Assessment Report of the Intergovernmental Panel on Climate Change* 923–1054 (Cambridge University Press, 2021). doi:10.1017/9781009157896.009.
25. Myhre, G. *et al. Anthropogenic and Natural Radiative Forcing. In: Climate Change 2013: The Physical Science Basis. Contribution of Working Group I*. (2013).
26. Bond, T. C., Zarzycki, C., Flanner, M. G. & Koch, D. M. Quantifying immediate radiative forcing by black carbon and organic matter with the Specific Forcing Pulse. *Atmos. Chem. Phys.* 11, 1505–1525 (2011).
27. Aamaas, B., Peters, G. P. & Fuglestvedt, J. S. A synthesis of climate-based emission metrics with applications Earth System Dynamics Discussions A synthesis of climate-based emission metrics with applications A synthesis of climate-based emission metrics with applications. *Earth Syst. Dynam. Discuss* 3, 871–934 (2012).
28. Joos, F. *et al.* Carbon dioxide and climate impulse response functions for the computation of greenhouse gas metrics: A multi-model analysis. *Atmos. Chem. Phys.* 13, 2793–2825 (2013).
29. Ward, D. S. *et al.* The changing radiative forcing of fires: Global model estimates for past, present and future. *Atmos. Chem. Phys.* 12, 10857–10886 (2012).
30. Meinshausen, M., Raper, S. C. B. & Wigley, T. M. L. Emulating coupled atmosphere-ocean and carbon cycle models with a simpler model , MAGICC6 – Part 1 : Model description and calibration. *Atmos. Chem. Phys.* 1417–1456 (2011) doi:10.5194/acp-11-1417-2011.
31. Breiman, L. Random Forests. *Mach. Learn.* 45, 5–32 (2001).
32. Solvik, K. K. *et al.* ABoVE: MODIS-Derived Daily Mean Blue Sky Albedo for Northern North America, 2000-2017. *ORNL DAAC, Oak Ridge, Tennessee, USA.* https://doi.org/https://doi.org/10.3334/ORNLDAAC/1605 (2019) doi:https://doi.org/10.3334/ORNLDAAC/1605.

33. Schaaf, C., Wang, Z. MCD43A1 MODIS/Terra+Aqua BRDF/Albedo Model Parameters Daily L3 Global - 500m V006 [Data set]. *NASA EOSDIS Land Processes DAAC.* https://doi.org/10.5067/MODIS/MCD43A1.006 (2015).

34. Pedregosa, F. *et al. Scikit-Learn: Machine Learning in Python Gaël Varoquaux Bertrand Thirion Vincent Dubourg Alexandre Passos PEDREGOSA, VAROQUAUX, GRAMFORT ET AL. Matthieu Perrot*. *Journal of Machine Learning Research* vol. 12 http://scikit-learn.sourceforge.net. (2011).

35. Hengl, T. *et al.* SoilGrids250m: Global gridded soil information based on machine learning. *PLoS One* 12, (2017).

36. Danielson, J. J. & Gesch, D. B. *Global Multi-Resolution Terrain Elevation Data 2010 (GMTED2010)*. (2011).

37. Gruber, S. Derivation and analysis of a high-resolution estimate of global permafrost zonation. *Cryosphere* 6, 221–233 (2012).

38. Mahony, C. R., Wang, T., Hamann, A. & Cannon, A. J. A global climate model ensemble for downscaled monthly climate normals over North America. *International Journal of Climatology* 42, 5871–5891 (2022).

39. Virkkala, A.-M. *et al.* Machine learning-based Arctic-boreal terrestrial ecosystem CO2 fluxes, 2001-2020. *ORNL DAAC, Oak Ridge, Tennessee, USA.* https://doi.org/https://doi.org/10.3334/ORNLDAAC/2377 (2024) doi:https://doi.org/10.3334/ORNLDAAC/2377.

40. Pallandt, M. M. T. A. *et al.* High-Latitude Eddy Covariance Temporal Network Design and Optimization. *J. Geophys. Res. Biogeosci.* 129, (2024).

41. Virkkala, A.-M. *et al.* Wildfires offset the increasing but spatially heterogeneous Arctic–boreal CO2 uptake. *Nat. Clim. Chang.* https://doi.org/10.1038/s41558-024-02234-5 (2025) doi:10.1038/s41558-024-02234-5.

42. Turetsky, M. R. *et al.* Carbon release through abrupt permafrost thaw. *Nat. Geosci.* 13, 138–143 (2020).

43. Gibson, C. M. *et al.* Wildfire as a major driver of recent permafrost thaw in boreal peatlands. *Nat. Commun.* 9, (2018).

44. Zhu, X., Xu, X. & Jia, G. Recent massive expansion of wildfire and its impact on active layer over pan-Arctic permafrost. *Environmental Research Letters* 18, (2023).

45. Schuur, E. A. G. *et al.* Climate change and the permafrost carbon feedback. *Nature* vol. 520 171–179 Preprint at https://doi.org/10.1038/nature14338 (2015).

46. Natali, S. M. *et al.* Large loss of CO2 in winter observed across the northern permafrost region. *Nat. Clim. Chang.* 9, 852–857 (2019).

47. Heffernan, L. *et al.* High peatland methane emissions following permafrost thaw: enhanced acetoclastic methanogenesis during early successional stages. *Biogeosciences* 19, 3051–3071 (2022).

48. Estop-Aragonés, C. *et al.* Limited release of previously-frozen C and increased new peat formation after thaw in permafrost peatlands. *Soil Biol. Biochem.* 118, 115–129 (2018).

49. Estop-Aragonés, C. *et al.* Respiration of aged soil carbon during fall in permafrost peatlands enhanced by active layer deepening following wildfire but limited following thermokarst. *Environmental Research Letters* 13, (2018).
